# Bilateral peripheral CT venography for evaluating central venous lesions in dysfunctional arteriovenous fistulas: feasibility, safety, and radiation dose in hemodialysis patients

**DOI:** 10.1080/0886022X.2026.2740365

**Published:** 2026-10-20

**Authors:** Hongwei Zhang, Hongming Liu, Zhi Zou, Shunqiang Chen, Lili Shi, Yongli Li, Shewei Dou

**Affiliations:** Department of Medical Imaging, Henan Provincial People’s Hospital, Zhengzhou, China

**Keywords:** Arteriovenous fistula, central venous stenosis, CT venography, hemodialysis, vascular access

## Abstract

**Objectives:**

Central venous stenosis and occlusion are major causes of arteriovenous fistula (AVF) dysfunction in hemodialysis patients. This study aimed to evaluate the feasibility, safety, radiation exposure, and anatomical findings of bilateral peripheral CT venography for central venous assessment in patients with dysfunctional AVFs.

**Methods:**

This single-center retrospective cohort study included 46 consecutive hemodialysis patients with dysfunctional AVFs who underwent bilateral peripheral CT venography between August 2023 and June 2025. Technical success, procedure-related adverse events, radiation dose parameters, central venous lesion characteristics, and agreement with available comparative imaging modalities were analyzed. Comparative imaging findings from puncture-based angiography and/or duplex ultrasonography were assessed descriptively.

**Results:**

All 46 examinations were technically successful (100%), with a mean procedure duration of 4 minutes (range, 3–5 minutes). Minor adverse events included one patient of contrast extravasation (2.2%) and 3 of mild allergic reactions (6.5%); no severe procedure-related complications were documented. The mean CTDIvol and DLP were 11.87 mGy and 784.15 mGy·cm, respectively. Central venous abnormalities were identified in 44 patients (95.7%), including stenosis in 31 (67.4%), occlusion in 11 (23.9%), and aneurysmal changes in 2 (4.3%). Comparative imaging was available in 38 patients, and CT venography showed agreement with available findings in 37 cases (97.4%). This agreement was interpreted as descriptive concordance rather than formal diagnostic accuracy.

**Conclusions:**

Bilateral peripheral CT venography demonstrated high technical feasibility, acceptable safety, and effective visualization of central venous abnormalities in hemodialysis patients with dysfunctional AVFs. Further prospective studies are needed to validate its diagnostic performance and clinical impact.

**Advances in knowledge:**

This technique enables efficient, noninvasive assessment of central venous pathology with minimal risk, supporting its potential as a practical alternative to conventional imaging in hemodialysis patients.

## Introduction

Maintaining reliable vascular access is essential for patients undergoing long-term hemodialysis. Arteriovenous fistulas (AVFs) are generally preferred over central venous catheters or prosthetic grafts because of their favorable long-term patency, lower infection risk, and reduced access-related morbidity [1]. However, AVF dysfunction remains a frequent clinical problem, resulting in inadequate dialysis delivery, prolonged bleeding after dialysis, arm swelling, and repeated interventions [2]. Central venous abnormalities, particularly central venous stenosis and occlusion, represent important causes of AVF dysfunction and are frequently associated with previous central venous catheterization or other vascular interventions [3]. Catheter-related endothelial injury, thrombosis, and subsequent fibrotic remodeling have been proposed as potential mechanisms contributing to these lesions. Early identification and anatomical characterization of these lesions are essential for guiding subsequent management strategies, including endovascular intervention and preservation of vascular access [4].

Current imaging approaches for evaluating central venous lesions in patients with dysfunctional AVFs have important limitations. Duplex ultrasonography is widely available, noninvasive, and commonly used as an initial assessment tool; however, its ability to evaluate deep central veins may be limited by anatomical factors such as the clavicle, ribs, and overlying air-containing structures, and its diagnostic performance is highly operator dependent [5,6]. Digital subtraction angiography is frequently used for detailed vascular assessment and procedural planning, but it requires invasive vascular access, may involve substantial contrast administration, and carries procedure-related risks, which are particularly relevant in patients with end-stage renal disease (ESRD) [7–9]. Conventional CT venography provides comprehensive cross-sectional visualization of central venous anatomy but may require relatively large contrast volumes and radiation exposure, limiting its routine application in dialysis-dependent populations [10].

Bilateral peripheral CT venography, using simultaneous low-volume contrast injection through peripheral forearm veins, has emerged as a potential alternative approach for evaluating central venous abnormalities in hemodialysis patients. By initiating contrast delivery from the extremities and incorporating optimized acquisition strategies, this technique may provide adequate visualization of central venous structures while reducing contrast burden compared with conventional approaches [11,12]. However, evidence regarding its technical feasibility, procedural safety, radiation exposure, and relationship with available clinical imaging assessments in patients with dysfunctional AVFs remains limited.

Therefore, this retrospective study aimed to evaluate the feasibility, safety profile, radiation exposure, and anatomical findings of bilateral peripheral CT venography in hemodialysis patients with dysfunctional AVFs. In addition, we explored its agreement with available comparative imaging modalities and assessed its potential clinical value for characterizing central venous lesions.

## Methods

### Study design and population

This was a single-center retrospective cohort study conducted at Henan Provincial People’s Hospital between August 2023 and June 2025. The study aimed to evaluate the technical feasibility, procedural safety, radiation exposure, and anatomical characterization capability of bilateral peripheral CT venography in hemodialysis patients with dysfunctional AVFs.

Consecutive adult patients with ESRD undergoing maintenance hemodialysis who were referred for imaging evaluation because of suspected central venous abnormalities were retrospectively identified. Clinical indications included reduced AVF flow, elevated venous dialysis pressures, arm swelling, prolonged bleeding after dialysis, or other physical examination findings suggestive of central venous stenosis or occlusion. AVF dysfunction was defined according to clinical criteria, including reduced dialysis adequacy (*Kt*/*V* < 1.2), elevated venous pressure (>200 mmHg), or abnormal findings during access assessment.

The inclusion criteria were: (1) age ≥18 years; (2) established AVF or arteriovenous graft for hemodialysis access; (3) stage 5 chronic kidney disease requiring maintenance dialysis; and (4) completion of bilateral peripheral CT venography. Patients were excluded if they had: (1) contraindications to iodinated contrast administration that precluded imaging; (2) inability to complete CT examination; or (3) incomplete imaging or clinical information.

A total of 46 patients fulfilled the eligibility criteria and were included in the final analysis.

The study protocol was approved by the Institutional Review Board of Henan Provincial People’s Hospital. Because of the retrospective design and use of routinely collected clinical imaging data, the requirement for informed consent was waived. All procedures were conducted in accordance with the Declaration of Helsinki.

### Imaging protocol

All CT venography examinations were performed using a standardized bilateral peripheral venography protocol on a GE Revolution CT scanner (GE Healthcare, Milwaukee, WI).

Patients were positioned supine with both upper limbs extended alongside the body. Bilateral antecubital venous access was obtained using 20-gauge intravenous catheters, which served as the injection sites and the initial points of venous opacification.

Iohexol (350 mgI/mL; Omnipaque, GE Healthcare, Milwaukee, WI) was diluted with normal saline at a ratio of 1:5. Contrast medium was administered simultaneously through both antecubital veins using a dual-head power injector at a rate of 2 mL/s per side. The total contrast volume was 40 mL per arm (80 mL in total).

CT acquisition was performed using helical mode with coverage from the upper extremities through the thoracic inlet and central veins. Imaging parameters included a tube voltage of 120 kV, automatic tube current modulation (100–300 mAs), pitch of 0.984, rotation time of 0.5 s, acquisition slice thickness of 5 mm, and reconstruction thickness of 1.25 mm.

To minimize artifacts caused by heterogeneous contrast mixing, the first-phase acquisition was synchronized to complete at the end of contrast injection, followed immediately by a delayed-phase acquisition. Radiation exposure parameters, including CT dose index volume (CTDIvol) and dose-length product (DLP), were automatically recorded from the CT system. Radiation exposure was interpreted in the context of CT venography protocols rather than routine diagnostic chest CT examinations because acquisition coverage and imaging objectives differ substantially between these examinations.

### Image interpretation and reference imaging assessment

All CT venography images were independently reviewed by two cardiovascular radiologists with more than 10 years of experience in vascular imaging. The reviewers were blinded to the findings of comparative imaging examinations, including duplex ultrasonography and puncture-based angiography, when interpreting CT venography images. Disagreements were resolved through consensus review.

Central venous abnormalities were assessed in the subclavian veins, brachiocephalic (innominate) veins, superior vena cava, and related venous junctions.

Where available, previous or concurrent reference imaging examinations were collected from medical records, including duplex ultrasonography and puncture-based angiography. Because these examinations were obtained as part of routine clinical care rather than according to a predefined diagnostic accuracy protocol, the reference modalities were not considered interchangeable. Therefore, agreement between CT venography and available comparative imaging was analyzed descriptively rather than as a formal diagnostic accuracy assessment.

The concordance analysis was based on agreement regarding the principal central venous finding (presence/location of clinically relevant abnormality). Sensitivity, specificity, positive predictive value, and negative predictive value were not calculated because a uniform reference standard was unavailable.

### Assessment of clinical impact

Clinical information regarding subsequent management after CT venography was retrospectively reviewed when available. In cases with documented follow-up, CT venography findings were used as an anatomical reference for vascular access planning and selection of subsequent interventions. Potential clinical impact included additional procedural planning, identification of lesion location relevant to vascular access management, or modification of subsequent interventions.

Because treatment decisions were influenced by multiple clinical factors and were not prospectively recorded as a predefined endpoint, the study did not attempt to quantify the independent causal effect of CT venography on therapeutic strategy.

### Data collection and statistical analysis

Clinical variables, including age, sex, ESRD duration, underlying renal disease, comorbidities, prior vascular interventions, AVF characteristics, and procedural parameters, were extracted from electronic medical records.

Information regarding previous central venous catheterization was reviewed when documented. However, because catheter-related information was incompletely available in this retrospective dataset, formal analyses evaluating the association between prior catheter placement and central venous lesions were not performed.

Procedural outcomes, including technical success, examination duration, adverse events, radiation dose parameters, lesion characteristics, and comparison with available imaging modalities, were summarized descriptively.

Continuous variables were presented as mean ± standard deviation with range, and categorical variables were presented as frequencies and percentages. Exploratory comparisons of radiation exposure parameters were performed using independent-samples *t*-tests or Pearson’s correlation analyses where appropriate. Given the limited sample size, subgroup analyses and regression models were considered exploratory and hypothesis-generating rather than confirmatory.

Agreement between CT venography and available comparative imaging was reported as percentage agreement with 95% confidence intervals. Statistical analyses were performed using GraphPad Prism version 10.0 (San Diego, CA) and Python 3.12 with SciPy and Statsmodels libraries. A two-sided *p* value <0.05 was considered statistically significant where applicable.

### Diagnostic criteria for central venous lesions

Central venous lesions were evaluated in the subclavian veins, brachiocephalic (innominate) veins, superior vena cava, and related junctions.

Significant stenosis was defined as >50% reduction in luminal diameter compared with an adjacent normal venous segment on multiplanar reconstruction and maximum intensity projection images. Severe stenosis was defined as ≥70% narrowing or near-occlusive appearance. Occlusion was diagnosed when complete absence of contrast opacification and interruption of venous continuity were observed.

Aneurysmal changes were defined as focal venous dilatation exceeding 150% of the expected adjacent normal venous diameter or showing irregular contour suggestive of pseudoaneurysm. Patent veins were defined as continuous contrast opacification without significant narrowing or filling defects.

Delayed-phase images were reviewed to distinguish true venous abnormalities from transient contrast mixing artifacts.

## Results

### Patient demographics and baseline characteristics

A total of 46 hemodialysis patients with dysfunctional AVFs underwent bilateral peripheral CT venography and were included in the analysis. The cohort comprised 25 men (54.3%) and 21 women (45.7%), with a mean age of 55.9 ± 12.6 years (range, 30–78 years). The mean duration of ESRD/dialysis was 7.1 ± 4.8 years (range, 1.0–19.2 years).

The underlying etiology of kidney disease was not specifically documented in 41 patients (89.1%). Among the remaining patients, the recorded etiologies were hypertensive nephropathy in two (4.3%), glomerulonephritis in one (2.2%), nonspecific renal insufficiency in one (2.2%), and diabetic nephropathy in one (2.2%). Hypertension was the most common comorbidity (42/46, 91.3%), followed by diabetes mellitus (12/46, 26.1%). Other recorded comorbidities included coronary heart disease (*n* = 2, 4.3%), heart failure (*n* = 1, 2.2%), hepatitis B (*n* = 1, 2.2%), other cardiac disease (*n* = 1, 2.2%), and cerebral infarction (*n* = 1, 2.2%). Four patients (8.7%) had no documented comorbidity.

A history of previous vascular intervention, including angioplasty or prior AVF-related procedures, was documented in 5 patients (10.9%). Previous central venous catheterization history was incompletely recorded in the retrospective dataset. Therefore, although central venous stenosis and occlusion identified on CT venography were consistent with previously reported catheter-associated complications, a patient-level analysis directly linking prior catheter placement with lesion location or severity could not be reliably performed.

All patients had established AVFs for hemodialysis access. Forty-four AVFs (95.7%) were autologous and two (4.3%) were prosthetic grafts. The most frequent access configuration was a left radiocephalic AVF (*n* = 23, 50.0%), followed by a right radiocephalic AVF (*n* = 12, 26.1%), left brachiobasilic AVF at the elbow (*n* = 6, 13.0%), left brachiobasilic AVF at another recorded location (*n* = 2, 4.3%), left radiobasilic AVF (*n* = 1, 2.2%), left cephalic AVF with a forearm arterial anastomosis (*n* = 1, 2.2%), and right brachiobasilic AVF at the elbow (*n* = 1, 2.2%). The mean AVF age was 5.4 ± 4.0 years (range, 0.7–17.3 years). Forty-four AVFs (95.7%) were mature and two (4.3%) were immature. Baseline characteristics are summarized in Table 1.

**Table 1.** Patient demographics and baseline characteristics.

Characteristic	Value
Total patients, *n*	46
Age, years (mean ± SD; range)	55.9 ± 12.6 (30–78)
Sex, *n* (%)	
Male	25 (54.3)
Female	21 (45.7)
ESRD/dialysis duration, years (mean ± SD; range)	7.1 ± 4.8 (1.0–19.2)
Underlying kidney disease, *n* (%)	
Not specifically documented	41 (89.1)
Hypertensive nephropathy	2 (4.3)
Glomerulonephritis	1 (2.2)
Nonspecific renal insufficiency	1 (2.2)
Diabetic nephropathy	1 (2.2)
Comorbidities, *n* (%)	
Hypertension	42 (91.3)
Diabetes mellitus	12 (26.1)
Coronary heart disease	2 (4.3)
Heart failure	1 (2.2)
Hepatitis B infection	1 (2.2)
Other cardiac disease	1 (2.2)
Cerebral infarction	1 (2.2)
No documented comorbidity	4 (8.7)
Previous vascular intervention, n (%)	5 (10.9)
AVF type, *n* (%)	
Autologous AVF	44 (95.7)
Prosthetic graft	2 (4.3)
AVF location, *n* (%)	
Left radiocephalic AVF	23 (50.0)
Right radiocephalic AVF	12 (26.1)
Left brachiobasilic AVF (elbow)	6 (13.0)
Left brachiobasilic AVF	2 (4.3)
Left radiobasilic AVF	1 (2.2)
Left cephalic AVF with forearm artery anastomosis	1 (2.2)
Right brachiobasilic AVF (elbow)	1 (2.2)
AVF age, years (mean ± SD; range)	5.4 ± 4.0 (0.7–17.3)
AVF maturity status, *n* (%)	
Mature	44 (95.7)
Immature	2 (4.3)

Data regarding previous central venous catheterization were not consistently available in the retrospective records and therefore were not included as a baseline variable.

### Technical feasibility

Bilateral peripheral CT venography was technically completed in all 46 patients, corresponding to a technical success rate of 100%. The mean procedure duration was 4 min (range, 3–5 min). All examinations were performed using the planned bilateral peripheral venous injection approach, and no patient required conversion to an alternative venous access site or termination of the examination because of technical failure.

No unsuccessful venous cannulation, equipment-related interruption, or incomplete CT acquisition was documented. The technical parameters used for image acquisition are summarized in Table 2.

**Table 2.** Technical parameters and feasibility of bilateral peripheral CT venography.

Parameter	Value
Total examinations, *n*	46
Technical success rate, *n* (%)	46 (100)
Injection approach	
Bilateral antecubital venous injection	46 (100)
Conversion to alternative access site	0
Procedure duration, min (mean, range)	4 (3–5)
Contrast protocol	
Contrast agent	Iohexol (350 mgI/mL)
Contrast dilution	1:5 with normal saline
Injection method	Simultaneous bilateral injection
Injection rate	2 mL/s per side
Contrast volume per arm	40 mL
Total contrast volume	80 mL
CT acquisition parameters	
CT scanner	GE Revolution CT scanner
Acquisition mode	Helical scanning
Tube voltage	120 kVp
Tube current modulation	Automatic (100–300 mAs)
Pitch	0.984
Rotation time	0.5 s
Acquisition slice thickness	5 mm
Reconstruction thickness	1.25 mm
Scanning strategy	
First-phase acquisition	Completed at the end of contrast injection
Delayed-phase acquisition	Immediately performed after first phase
Purpose of delayed phase	Reduction of false-positive findings caused by contrast mixing artifacts
Technical failures, *n* (%)	
Failed venous cannulation	0 (0)
Equipment-related interruption	0 (0)
Incomplete CT acquisition	0 (0)

### Safety and procedure-related events

The procedure was generally well tolerated. Contrast extravasation occurred in one of 46 patients (2.2%), and mild allergic symptoms, including urticaria or pruritus, were documented in three patients (6.5%). These events resolved without major intervention. No severe anaphylactic reaction, procedure-related hospitalization, or procedure-related death was recorded.

No puncture-site infection, clinically documented vascular injury, or delayed hypersensitivity reaction was identified in the available follow-up records. Because systematic pre- and post-examination renal biochemical measurements and dialysis-adequacy data were not available, contrast-associated changes in renal function could not be objectively assessed. Accordingly, the present analysis was limited to clinically documented contrast-related adverse events and did not permit a formal determination of the incidence of contrast-associated acute kidney injury.

Radiation exposure was recorded for all examinations and is reported below. No clinically apparent radiation-related adverse event was documented during the available follow-up period. Procedure-related safety findings are summarized in Table 3.

**Table 3.** Procedure-related safety events and adverse outcomes after bilateral peripheral CT venography.

Safety event	Incidence, *n* (%)	Description
Total patients	46	
Contrast-related events		
Contrast extravasation	1 (2.2)	Minor leakage at injection site; resolved without major intervention
Mild allergic reaction	3 (6.5)	Urticaria or pruritus; resolved without major intervention
Severe anaphylactic reaction	0 (0)	None documented
Procedure-related complications		
Failed venous cannulation	0 (0)	All examinations completed successfully
Procedure-related vascular injury	0 (0)	None documented
Puncture-site infection	0 (0)	None documented during available follow-up
Delayed hypersensitivity reaction	0 (0)	None documented
Procedure-related hospitalization	0 (0)	None documented
Procedure-related mortality	0 (0)	None documented
Renal safety assessment		
Contrast-associated acute kidney injury	Not assessable	Systematic pre- and post-examination renal biochemical measurements were unavailable
Dialysis after CT venography	46 (100)	Routine dialysis performed after examination according to clinical practice
Radiation-related adverse events		
Clinically apparent radiation-related complications	0 (0)	None documented during available follow-up

Values are presented as number (%). Contrast-associated acute kidney injury was not formally assessed because systematic renal biochemical measurements before and after CT venography were unavailable.

### Central venous findings on CT venography

Bilateral peripheral CT venography demonstrated a central venous abnormality in 44 of 46 patients (95.7%). This high proportion should be interpreted in the context of the study population, which consisted of patients referred because of clinically suspected AVF dysfunction.

Stenosis was identified in 31 patients (67.4%), occlusion in 11 (23.9%), and aneurysmal change in two (4.3%). The remaining two patients (4.3%) had patent central veins without a significant abnormality. Among the 31 patients with stenosis, 20 (64.5%) were classified as having severe/high-grade stenosis, one (3.2%) as having mild-to-moderate stenosis, and 10 (32.3%) did not have sufficiently detailed retrospective documentation to permit reliable severity classification.

With respect to laterality, abnormalities were recorded on the left side in 27 patients (58.7%), on the right side in 12 (26.1%), and bilaterally in one (2.2%); laterality was not specifically recorded in six cases (13.0%). Because lesion location could involve more than one contiguous central venous segment, anatomical site categories were not mutually exclusive.

The innominate/brachiocephalic vein was the most frequently involved central venous segment, either as an isolated lesion or in combination with adjacent venous structures. Subclavian venous involvement was also common, including lesions extending to or from the innominate vein. Less frequently involved sites included other cephalic/brachiocephalic venous segments and the superior vena cava junction. Detailed distributions according to lesion type, anatomical location, laterality, and stenosis severity are provided in Table 4.

**Table 4.** Distribution of central venous findings identified by bilateral peripheral CT venography.

Finding	*n* (%)
Total patients	46 (100)
Overall central venous findings	
Central venous abnormality identified	44 (95.7)
No significant central venous abnormality (patent veins)	2 (4.3)
Type of central venous lesion	
Stenosis (>50% luminal narrowing)	31 (67.4)
Occlusion	11 (23.9)
Aneurysmal change	2 (4.3)
Patent central veins	2 (4.3)
Stenosis severity (among stenosis cases, *n* = 31)	
Severe/high-grade stenosis (≥70% narrowing or near-occlusive)	20 (64.5)
Mild-to-moderate stenosis (<70%)	1 (3.2)
Severity not reliably classified	10 (32.3)
Laterality of central venous abnormalities	
Left-sided involvement	27 (58.7)
Right-sided involvement	12 (26.1)
Bilateral involvement	1 (2.2)
Laterality not specified	6 (13.0)
Anatomical distribution of involved venous segments*	
Anatomical location	*n* (%)
Innominate/brachiocephalic vein	33 (71.7)
Subclavian vein	14 (30.4)
Superior vena cava (SVC) or SVC junction	2 (4.3)
Cephalic/brachiocephalic venous segment	3 (6.5)
Other central venous segments	7 (15.2)

*Note*: *P < 0.05.

### Radiation exposure and exploratory associations

Radiation-dose analyses were considered exploratory because of the modest sample size. Mean CTDIvol was 11.92 ± 0.74 mGy in men and 11.81 ± 0.73 mGy in women, with no statistically significant difference between the groups (*t* = 0.54, *p* = 0.590). Mean DLP was 797.5 ± 84.2 mGy·cm in men and 767.8 ± 79.6 mGy·cm in women (*t* = 1.25, *p* = 0.218).

No statistically significant linear correlation was observed between age and CTDIvol (*r* = −0.05, *p* = 0.754) or DLP (*r* = −0.08, *p* = 0.602). Similarly, ESRD/dialysis duration was not significantly correlated with CTDIvol (*r* = 0.03, *p* = 0.842) or DLP (*r* = 0.06, *p* = 0.698) (Table 5). Because only 21 of the 31 stenotic lesions had an explicitly documented severity category and only one of these was classified as mild-to-moderate, formal subgroup comparisons and multivariable modeling of severe stenosis were not considered sufficiently robust and were therefore not used to support inferential conclusions.

**Table 5.** Exploratory analysis of radiation exposure parameters.

Variable	CTDIvol (mGy)	*p* Value	DLP (mGy·cm)	*p* Value
Sex				
Male (*n* = 25)	11.92 ± 0.74	0.59	797.5 ± 84.2	0.218
Female (*n* = 21)	11.81 ± 0.73		767.8 ± 79.6	
Correlation analysis				
Age (Pearson’s *r*)	−0.05	0.754	−0.08	0.602
ESRD/dialysis duration (Pearson’s *r*)	0.03	0.842	0.06	0.698

### Comparison with other imaging modalities

Comparative imaging information was available for 38 of the 46 patients (82.6%), including puncture-based angiography in 24 patients and duplex ultrasonography in 22 patients (Table 6). Among these patients, 8 patients underwent both modalities; therefore, the categories were not mutually exclusive. CT venography and the available comparison modality showed agreement regarding the principal central venous finding in 37 cases (97.4%; 95% CI, 86.2%–99.9%). The single discordant case involved a difference in stenosis severity grading rather than the presence versus absence of a central venous lesion.

**Table 6.** Comparison between bilateral peripheral CT venography and available reference imaging modalities.

Parameter	Value
Total patients undergoing CT venography	46
Patients with available comparative imaging	38 (82.6%)
Patients without comparative imaging	8 (17.4%)
Reference imaging modalities*	
Puncture-based angiography	24 (63.2%)
Duplex ultrasonography	22 (57.9%)
Both modalities performed	8 (21.1%)
Agreement with CT venography	
Agreement regarding principal central venous finding	37 (97.4%)
Discordant cases	1 (2.6%)
Nature of discrepancy	Difference in stenosis severity grading only

*Note*: *P < 0.05.

This percentage was treated as a descriptive measure of agreement rather than a formal estimate of diagnostic accuracy. Sensitivity, specificity, positive predictive value, and negative predictive value were not calculated because comparative imaging was unavailable in eight patients, the reference modalities were heterogeneous, and the study cohort was selectively enriched for patients with clinically suspected AVF dysfunction.

Accordingly, the observed agreement supports the feasibility of bilateral peripheral CT venography for characterizing central venous abnormalities in this selected clinical cohort but does not establish its sensitivity or specificity relative to a uniform reference standard.

### Relationship of CT venography findings to clinical management

CT venography provided anatomical information on the presence, location, extent, and severity of central venous abnormalities in patients undergoing evaluation for AVF dysfunction. However, because this was a retrospective study, subsequent therapeutic decisions were not systematically recorded in a manner that allowed changes in treatment strategy to be attributed specifically to the CT venography findings rather than to the overall clinical and imaging assessment.

However, because treatment decisions incorporated multiple factors, including clinical symptoms, dialysis access function, and findings from other imaging examinations, the independent contribution of CT venography to therapeutic decision-making could not be isolated in this retrospective cohort. The present results consequently support the technical and anatomical utility of the examination but do not establish a direct causal effect on subsequent treatment selection.

## Discussion

This study evaluated the feasibility, safety profile, radiation exposure, and anatomical characterization capability of bilateral peripheral CT venography in hemodialysis patients with dysfunctional AVFs. The technique achieved a technical success rate of 100%, with a mean examination duration of approximately 4 min, demonstrating its practical applicability in routine clinical workflows. The standardized protocol using simultaneous bilateral antecubital venous injection of diluted iohexol enabled visualization of the peripheral and central venous pathways while maintaining a relatively low contrast volume. These findings are consistent with previous reports describing the feasibility of CT-based vascular access evaluation in patients undergoing hemodialysis [13,14].

The safety profile observed in this cohort was favorable. Only minor procedure-related events were documented, including contrast extravasation and mild allergic reactions, and no severe adverse events requiring intervention occurred. Compared with conventional catheter-based venography or interventional angiographic procedures, peripheral CT venography avoids additional invasive vascular access and may reduce procedure-related risks associated with catheter manipulation [15,16]. However, because systematic biochemical monitoring before and after imaging was not available, the present study assessed only clinically documented adverse events and did not directly evaluate contrast-associated acute kidney injury. In dialysis-dependent patients, contrast clearance through subsequent dialysis may reduce concerns regarding prolonged contrast exposure, but this should not be interpreted as evidence of complete elimination of renal-related risks [17].

Radiation exposure was relatively low, with mean CTDIvol of 11.87 mGy and DLP of 784.15 mGy·cm. These values were obtained using a standardized acquisition protocol incorporating optimized scanning parameters and limited acquisition coverage. Because CTDIvol values are highly dependent on scan coverage, acquisition parameters, and clinical purpose, direct comparison with routine chest CT protocols is inappropriate. The observed radiation exposure was comparable to previously reported optimized CT venography protocols, supporting the feasibility of dose reduction strategies for vascular access imaging [18]. Continued attention to dose optimization remains important, particularly in patients requiring repeated vascular access evaluations.

Bilateral peripheral CT venography identified central venous abnormalities in 44 of 46 patients (95.7%), including stenosis, occlusion, and aneurysmal changes. This high prevalence should be interpreted in the context of the study population, which consisted exclusively of patients referred for imaging because of clinically suspected AVF dysfunction. Therefore, these findings reflect a selected high-risk cohort rather than the prevalence of central venous lesions among all hemodialysis patients. Because patients without suspected AVF dysfunction were not included, false-positive rates and specificity in lower-prevalence populations could not be assessed. Previous studies have reported that central venous abnormalities are important contributors to AVF dysfunction, particularly among patients with previous vascular interventions or catheter exposure [19]. Duplex ultrasonography remains an important first-line modality for preprocedural mapping, maturation assessment, and evaluation of AVF dysfunction; however, assessment of central venous segments may be limited by anatomical constraints and operator dependence [20]. In contrast, bilateral CT venography approaches using optimized contrast injection strategies have been reported to provide comprehensive visualization of vascular access anatomy, supporting the technical feasibility of CT-based evaluation in hemodialysis patients [21].

The comparison between CT venography and available imaging modalities demonstrated agreement in 37 of 38 cases (97.4%). However, this finding should be interpreted as descriptive concordance rather than formal diagnostic accuracy. Comparative imaging was not available in all patients, and the reference examinations consisted of heterogeneous modalities, including duplex ultrasonography and puncture-based angiography. Because these techniques have different acquisition principles and diagnostic capabilities, they cannot be considered equivalent reference standards. Furthermore, the study design did not allow calculation of sensitivity, specificity, or predictive values. Future prospective studies using a predefined reference standard are required to establish the diagnostic performance of bilateral peripheral CT venography.

The high lesion detection rate and anatomical information provided by CT venography may have potential clinical value for planning subsequent vascular access management. By demonstrating lesion location, extent, and relationship with adjacent venous structures, this technique may assist clinicians in selecting appropriate treatment strategies, including endovascular intervention. However, because treatment decisions in this retrospective cohort were influenced by multiple clinical factors and were not prospectively recorded, the independent contribution of CT venography to therapeutic decision-making could not be quantified.

In this study, radiation exposure parameters did not significantly differ according to sex, age, or ESRD duration. These exploratory findings describe radiation exposure patterns within this cohort; however, the limited sample size and absence of external validation prevent conclusions regarding the reproducibility of dose consistency in broader populations. Similarly, the available dataset was insufficient to determine reliable predictors of lesion severity, and exploratory subgroup analyses should be interpreted cautiously.

Several limitations should be acknowledged. First, this was a retrospective single-center study with a relatively small sample size, which limits generalizability. Second, because patients were selected based on clinical suspicion of AVF dysfunction, the high proportion of detected lesions may reflect selection bias and does not represent screening performance in an unselected dialysis population. Third, comparative imaging was available only in a subset of patients, and the reference modalities were heterogeneous, limiting formal evaluation of diagnostic accuracy. Fourth, previous central venous catheterization history was incompletely documented, preventing a robust analysis of its association with central venous lesions. Finally, long-term clinical outcomes, including changes in intervention strategy, AVF survival, and repeated access failure, were not systematically evaluated.

In conclusion, bilateral peripheral CT venography demonstrated high technical feasibility, acceptable procedural safety, and effective anatomical visualization of central venous abnormalities in hemodialysis patients with dysfunctional AVFs. The technique may serve as a useful noninvasive imaging approach for evaluating central venous anatomy in selected patients. Larger prospective multicenter studies with standardized reference imaging and longitudinal follow-up are warranted to further define its diagnostic performance and clinical impact.

## Data Availability

The datasets analyzed during the current study are not publicly available because they contain clinical information derived from individual patients. However, de-identified data may be available from the corresponding author upon reasonable request and subject to approval by the institutional review committee.
